# Investigation of the effects of mir-219-1 gene variants on the development of disease in non-small cell lung cancer patients

**DOI:** 10.4314/ahs.v22i4.6

**Published:** 2022-12

**Authors:** Sevgi Kalkanli Tas, Ender Coskunpinar, Pinar Yildiz, Mesut Bayraktaroğlu, Tuba Kose, Derya Altunkanat, Duygu Kirkik, Mustafa Tukenmez

**Affiliations:** 1 University of Health Sciences Turkey, Hamidiye Faculty of Medicine, Department of Immunology, Istanbul, Turkey; 2 University of Health Sciences Turkey, Hamidiye Faculty of Medicine, Department of Medical Biology, Istanbul, Turkey; 3 University of Health Sciences Turkey, Yedikule Chest Diseases and Chest Surgery Research and Training Hospital, Third Clinic, Istanbul, Turkey; 4 Istanbul University, Istanbul Faculty of Medicine, Department of General Surgery, Istanbul, Turkey

**Keywords:** NSCLC, miR-219-1 gene, single-nucleotide polymorphisms

## Abstract

**Background:**

Various variants of the miR-219-1 gene are one of the first genes associated with NSCLC prognosis in the literature.

**Objectives:**

We aimed to genotype two different variants of the miR-219-1 gene and to investigate to using of the result as a biomarker in the diagnosis and treatment of NSCLC.

**Materials and Methods:**

The patients were chosen according to International NSCLC criteria and genomic DNA was isolated from blood (138 patients and 100 healthy individuals). Then qRT-PCR was applied to determine the rs213210 and rs421446 variants of miR-219-1 gene polymorphisms. Allele and genotype frequencies were compared using Pearson's chi-square and Fisher's exact tests test.

**Results:**

We found that TT genotype (p=0,381) in rs213210 compared with CC genotype (p=0,165) and CC genotype (p=0,823) in rs421446 compared with TT genotype (p=0,537) did not show a significantly increased risk of NSCLC. There is no relationship between polymorphisms in miR-219-1 and the outcome of NSCLC.

**Conclusion:**

miRNA single nucleotide polymorphisms can be used as genetic biomarkers to predict cancer susceptibility, early diagnosis, and prognosis. Our study has shown that two variants of miR-219-1 were not related to NSCLC in the Turkish population. The reason for this can be differences in ethnicity, regions, and background of population and these differences could lead to various outcomes.

## Introduction

Non-small cell lung cancer (NSCLC) is known to be the most lethal cancer type among all cancers in the whole World and it contributes to 80–85% of lung cancers 1. There are several approaches for treatment including radiotherapy, chemotherapy, surgical resection and these modalities are also used as combinations. However, most of the patients (approximately 70%) exhibit weak responses to these treatments^2^. Therefore, it is a challenge for researchers to come up with new strategies in order to create an effective response to the treatment. In this regard, molecular approaches are promising for the better understanding of the mechanism and so it might help to create more effective therapeutic methods. According to 2019 data, lung cancer is the second most common type of cancer (13% of the 10 leading cancer types in the USA) with the highest mortality rate among men over 40 and women over 60 all around the world. According to GLOBOCAN's (Global Cancer Observatory) 2018 data, it is estimated that there are approximately 2 million new cases and 1.7–1.8 million deaths^3^,^4^. According to data distribution in Turkey, lung cancer is the most common type of cancer in males while fifthly in women, and the disease is more common in individuals between the ages of 55–74^5^,^6^. There are many possible risk factors for NSCLC, including radon, exposure to substances such as chromium, nickel, beryllium, asbestos, soot, or tar, air pollution, and family history of lung cancer^7^. More than 6000 components of cigarettes have potential of causing DNA damage which is known to be the primary underlying cause of cancer^8^. Although cells are using repair mechanisms to get over these DNA damages, the remaining unrepaired DNA damages from tobacco are likely to bring about NSCLC. Although cigarette consumption is the fundamental risk factor, recent studies showed that genetic variations might play a significant role in tumor development and progression^9^. Hence, determination of genetic biomarkers has potential to provide better risk prediction and improve clinical results of NSCLC patients^10^.

miRNAs, a type of non-coding RNAs, are approximately 20–25 nucleotides long, single-stranded, endogenous, short RNA sequences that do not encode proteins, acting as negative gene regulators at the transcriptional and posttranscriptional level. It has been suggested that dysregulation of miRNAs is directly or indirectly related to the pathogenesis of the disease in many types of cancer such as lung cancer. It is thought that the abnormal expression of miRNAs can trigger the onset and development of cancer, metastasis, and cause drug resistance by changing the expression of the genes it affects. As a result of studies conducted so far, a number of miRNAs thought to be associated with NSCLC have been revealed^11^–^13^. miR-138 is involved in the regulation of immune response, inhibition of cell proliferation and migration, as well as tumor suppression. miR-138-5p has been found to inhibit tumor growth and activate immunity by down-regulating PD-1 and PD-L1, making it a suitable therapeutic target for NSCLC^14^. It has been observed that miR-195-5p is abnormally expressed in many types of cancer. It has also been found that miR-195-5p can inhibit the proliferation of NSCLC cells and induce apoptosis by down-regulation of CEP55. In addition, it has been observed that miR-195-5p has a decreasing expression in NSCLC tissues and negatively affects the survival of the patients^15^. In a study that associated SNPs in miRNAs, miR-196a2 (rs11614913), miR-146a (rs2910164), miR-423 (rs6505162), miR-492 (rs2289030) and miR-27a (rs895819) pre-miRNAs and miR-26a-1 (rs7372209) and miR-219-1 (rs213210) pri-miRNA SNPs were examined. As a result of the study, it was determined that miR-146a (rs2910164) and miR-196a2 (rs11614913) variants were associated with NSCLC prognosis^16^. In another study on pri-miR-219-1 polymorphisms, rs213210, rs421446, and rs107822 variants, were taken from patients in terms of susceptibility to NSCLC and prognosis, and it was suggested that rs213210 and rs107822 variants could be genetic biomarkers for lung cancer risk^17^. It is expected that such molecular biomarkers will assist in the early diagnosis of lung cancer and the development of targeted therapy methods. In this study, we aimed to examine the rs213210 and rs421446 variants of miR-219-1 gene in NSCLC patients and first reveal the mechanisms in the pathways involved in the formation process of the disease and thus identify biomarker/s that can take place in a routine use.

## Materials and Methods

### Ethics approval

Ethical approval was taken from, Ethics Committee of the Istanbul Faculty of Medicine (Project No: 2017/034, Ethical Approval No: 849).

### Study population and collection of specimens

The study protocol was approved by the Ethics Committee of the Istanbul Faculty of Medicine (Project No: 2017/034, Ethical Approval No: 849). Informed consents were gotten from all NSCLC patients and control groups for our study. The number of volunteers were determined by power analysis (G*Power Statistical Power Analysis Program Ver. 3.1.9.4, Heinrich-Heine-University). 138 patients with NSCLC and 100 healthy individuals without lung diseases were included in our study who identified with the result of radiological and pathological examinations at the 3rd clinic of Yedikule Chest Diseases and Chest Surgery Training and Research Hospital. Our strict exclusion criteria for the study population were included those with primary extra-pulmonary malignancy, small cell lung cancer, a history of malignant disease, volunteer under 18 years of age, and the absence of consent of the patient.

### DNA isolation

2 ml of blood samples were obtained from the volunteers, and the samples were transported to the Department of Medical Biology, School of Medicine, University of Health Sciences by cold chain, and all samples were kept in a +4°C refrigerator for DNA isolation. DNA samples were gotten from leukocytes in venous blood. The manufacturer's instructions were followed for the DNA extraction (Product No: 11796828001 Roche Applied Sciences, Germany)^18^–^20^. After DNA isolation, the concentration and purity of the DNA samples were measured by spectrophotometer (Denovix DS-11 FX, USA), and obtained DNA samples were stored at -200C until further processing.

### Genotyping of rs213210 and rs421446 variants with Real-Time PCR

rs213210 (T>C) and rs421446 (C>T) variants of miR-219-1 gene polymorphism were analyzed by real-time PCR method. PCR components were adjusted so that the total volume was 20 µl for each sample according to manufacturer's instructions (LightCycler® FastStart DNA Master HybProbe, Cat. No. 03 003 248 001, V 16, Roche Diagnostics, Germany). PCR mix included PCR Grade Water, 13.5 µl; MgCl2 stock solution (25 µM), 1.5 µl; PCR Primer Mix (10x conc.), 2 µl; HybProbe Probe Mix (10x conc.), 2 µl; LightCycler® FastStart DNA Master HybProbe (10x conc.), 1 µl, and Template DNA 2 µl. Genotyping of rs 213210 and rs 421446 variants analyses were performed by Real-Time PCR and briefly, the conditions of PCR were as follows: Denaturation was at 95°C for 10 min, amplification was 45 cycles of 95°C for 10 s, annealing was at 60°C for 10 s and extension was 72°C for 10 s. Then, melting curve analysis was done at 95°C for 30 s, 40°C for 30 s, and 75°C for 0 s in order to detect non-specific amplifications. (Light Cycler® 480 Real-Time PCR System, Cat. No. 05015243001, Roche Applied Science, Germany)^21^,^22^.

### Statistical Analysis

Relevant gene regions that can play an active role in NSCLC formation, determined in terms of polymorphic and categorical data, were compared with student t-test. All statistical analyses were done by SPSS version 20.0 for Windows (SPSS Inc, Chicago, USA). Pearson's chi-square and Fisher's exact tests were used to analyze the differences in demographic variables such as smoking, alcohol consumption, and rs213210 and rs421446 variants of miR-219-1 genotype frequencies between patients and controls. The allele frequencies of rs213210 and rs421446 variants of miR-219-1 gene polymorphisms were calculated by Hardy- Weinberg equilibrium. Numeric values were examined by Student's t-test. A p-value of <0.05 was accepted as significant statistically.

## Results

The mean ages (±SD) of the NSCLC patient and control groups were 61 ± 9.50 and 53.89 ± 7.41 years, respectively. The gender distribution in NSCLC patients (93.48% male) was comparable with that in healthy controls (64% male, p-value: 0.0001), and the risk of NSCLC was increased 14.3-fold among male patients. The rates of smoking were 96.38% for the patient group (Table 1). Histology and clinic stage are also shown in Table 1. Genotype frequencies for miR-219-1 rs213210 and rs421446 patients and controls are listed in Table 2 and Table 3. The distribution of the miR-219-1 rs213210 and rs421446 genotypes and alleles in control and NSCLC patients was not found to be significantly different. The variant CC of miR-219-1 rs213210 was rare in patients 1.44 % (2/138) of patients, and this genotype was not found in the control group. The variant TT of miR-219-1 rs421446 was rare in patients 5.79 % (8/138) of patients, and 4 % of control group (4/100) (Table 2 and Table 3). Interaction of miR-219-1 variants and tobacco exposure on NSCLC were listed in Table 4. miR-219-1 SNPs associated with outcomes of NSCLC patients and stratified by stages are listed in Table 5 and Table 6.

**Table 1:** Demographics of NSCLC patients and controls

		Cases (n/%)	Controls (n/%)	P value
Gender	Male	129 (93,48)	66 (66)	*0,0001
	Female	9 (6,52)	34 (34)	
Age (years)	Mean ± SD	61±9,50	54,89±7,41	*0,005
Smoke (P/YEAR)	SMOKED	133 (96,38)	100 (%100)	0,076
	NON-SMOKED	5 (3,62)	0 (%0)	
	ADENO	11 (7,97)		
Histology	SQUA	32 (23,19)		
	NSCLC	25 (18,12)		
	Unknown	70 (50,72)		
Clinic stage	1A+1B	10 (7,25)		
	2A+2B+2	39 (28,26)		
	3A+3B+3	51 (36,96)		
	4	29 (21,01)		
	Unknown	9 (6,52)		

Pearson's chi-square and Fisher's exact tests were used.

Student's t test was applied.

**Table 2:** Distribution of miR-219-1 rs213210 genotypes and ORs for NSCLC cases and controls

Genotype	Cases	Controls	P-value	OR	95%CI	P adj	OR adj	95%CI adj
TT	118	80	0,381	Reference	Reference	0,417	Reference	Reference
TC	18	20	0,999	0,000	0,000	0,999	0,000	0,000
CC	2	0	0,165	0,000	0,816–3,291	0,186	1,692	0,776–3,687
TT								
TC+CC	20	20	0,206	0,699	0,401–1,220			
TT+TC	136	100		1,068	0,962–1,184			
CC								
T allele	254	180	0,441	1,023	0,965–1,083			
C allele	22	20		0,797	0,447–1,420			

P adj is adjusted by smoking, age and gender.

Student's t test was applied.

Allele frequencies were calculated with the Hardy-Weinberg balance.

**Table 3:** Distribution of miR-219-1 rs421446 genotypes and ORs for NSCLC cases and controls

Genotype	Cases	Controls	P-value	OR	95%CI	P adj	OR adj	95%CI adj
CC	75	55	0,823	Reference	Reference	0,942	Reference	Reference
CT	55	41	0,548	1,467	0,420–5,117	0,987	0,989	0,252–3,878
TT	8	4	0,537	1,491	0,420–5,290	0,868	0,890	0,224–3,540
CC								
CT+TT	63	45	0,888	1,023	0,746–1,401			
CC+CT	130	96		0,989	0,852–1,149			
TT								
C allele	205	151	0,181	1,069	0,968–1,181			
T allele	71	49		0,787	0,555–1,117			

P adj is adjusted by smoking, age and gender.

Student's t test was applied.

Allele frequencies were calculated with the Hardy-Weinberg balance

**Table 4:** Interaction of miR-219-1 variants and tobacco exposure on NSCLC.

Genotype	Smoking	Cases	Controls	P value	OR (95% CI)	P value adj.	OR (95% CI) adj.
**rs213210**							
TT	+	114	80	0,152	1,676 (0,827–3,399)	0,186	1,692 (0,776–3,687)
TC	+	17	20	0,999	0,000 (0,000)	0,999	0,000 (0,000)
CC	+	2	0	0,358	Reference	0,417	Reference
TT	-	4	0				
TC	-	1	0				
CC	-	0	0				
**rs421446**							
CC	+	72	55	0,904	Reference	0,942	Reference
CT	+	54	41	0,656	1,337 (0,373–4,797)	0,987	0,989
TT	+	7	4	0,667	1,329 (0,364–4,845)	0,868	0,890
CC	-	3	0				
CT	-	1	0				
TT	-	1	0				

P-value adj is adjusted by age and gender.

Student's t test was applied.

Pearson's chi-square and Fisher's exact tests were used.

Allele frequencies were calculated with the Hardy-Weinberg balance.

**Table 5:** miR-219-1 rs213210 SNP associated with outcomes of NSCLC patients and stratified by stages

Genotype	Cases	P-value	HR	95%CI
TT	118	0,487		
TC	18			
CC	2			
Stage I+II				
T allele	92	0,418	1,950	0,384–9,900
C allele	6		0,959	0,812–1,108
Stage III+IV				
T allele	144	0,125	2,464	0,732–8,290
C allele	16		0,920	0,836–1,012

P adj is adjusted by smoking, age and gender.

Student's t test was applied.

Pearson's chi-square and Fisher's exact tests were used.

Allele frequencies were calculated with the Hardy-Weinberg balance.

**Table 6:** miR-219-1 rs421446 SNP associated with outcomes of NSCLC patients and stratified by stages

Genotype	Cases	P-value	HR	95%CI
CC	75			
CT	55	0,001		
TT	8			
Stage I+II				
C allele	67			
T allele				
C allele	31	0,210	1,224	0,932–1,606
T allele			0,578	0,228–1,462
Stage III+IV				
C allele	125			
T allele				
C allele	35	0,502	0,944	0,800–1,113
T allele				

P adj is adjusted by smoking, age and gender.

Student's t test was applied.

Pearson's chi-square and Fisher's exact tests were used.

Allele frequencies were calculated with the Hardy-Weinberg balance.

## Discussion

Lung cancer is one of the most common malignancies in the world. Recently, genomic profile of lung cancer has been demonstrated and some tumors are genetically highly heterogeneous and complex^23^,^24^. The incidence and molecular structure of lung cancer differ between populations^25^,^26^. The NSCLC can remain undetected for years so that it is usually diagnosed in advanced stages. Thus, the success of treatment is extremely low 27. Many important evidences have shown that miRNAs and miRNA biogenesis mechanisms play a role in cancer development^27^,^28^. miRNAs are a type of small, endogenous, and single-stranded RNA molecules that have a role in the regulation of many cellular functions and are frequently dysregulated in various cancers due to polymorphisms and epigenetic alterations^13^,^29^,^30^. Mature miRNAs reduce the expression level of target genes and they participate in the regulation of protein synthesis. microRNAs have the ability to recognize target genes complementary to their nucleotide sequences. Following the discovery of their significance in carcinogenesis, many studies focused on miRNAs as potential circulating biomarkers for the early detection of cancers^31^. Also, a lot of studies have revealed their potential as circulating biomarkers for lung, breast, lymphoma, prostate, colorectal cancers, and glioblastomas^32^. Recently, a lot of miRNA polymorphisms have been found to change the regulatory role of their mRNA processing and gene interactions. miRNA Single Nucleotide Polymorphisms (SNPs) may be used as genetic markers to determine cancer susceptibility, early diagnosis, and prognosis^33^,^34^. Given that the development of different cancer types probably includes multiple stages, multigenic pathways, it is improbable that any single miRNA polymorphism would have a dramatic effect on the survival result. Thus, it is significant to conduct a pathway-based examination that assesses the combined effects of polymorphisms that may interact in the identical pathway. In the current study, we investigated the genotype frequencies and the clinical data from 138 patients to test the effects of 2 polymorphisms on miR219-1 gene for lung cancer risk. miR-219-1 was initially reported as a brain-specific miRNA. miR-219-1, which is located on chromosome 6p21.32. miR-219-1 is a light-and clock-regulated gene that controls the length of the daily (circadian) cycle as this miRNA is an important regulator of oligodendrocyte differentiation^35^,^36^. miR-219-1 rs213210 gene variant has considerably enhanced the mortality risk in stage III colorectal adenocarcinoma and patients with oesophageal cancer. These data have shown that miR-219-1 polymorphisms can promote the susceptibility of other cancer types but there is no information about the relationship between miR-219-1 polymorphism and NSCLC risk in the literature^34^,^37^. We are the first research group who investigated the effects of miR-219-1 gene variants on the risk factors of the disease in patients with NSCLC in Turkish Population. Peng et. al. has shown that the variant G allele of the rs2910164 locus was related to enhanced expression of miR-146a and to suppress breast cancer metastasis^38^. Moreover, the inhibitory effect of miR-146a on cancer invasion has also been reported in pancreatic cancer cells that enhanced expression epidermal growth factor receptor and its target, the nuclear factor-kB (NF-kB) regulatory kinase interleukin-1 receptor-associated kinase 1^34^. Song et al. have shown that there is an important relationship between the rs421446 polymorphism and non-small cell lung cancer prognosis in patients with advanced-stage disease in 2015^39^. In addition to this, our results are not similar to previous reports. The reason for this can be differences in ethnicity, regions, and background of population and these differences could lead to various outcomes.

## Conclusion

In conclusion, our study has shown that two variants of miR-219-1 (rs213210 and rs421446) were not related to NSCLC in the Turkish population. Our case-control study has some limitations. For example; our sample size was small to evaluate the relationship between the rs213210 and rs421446 variants of miR-219-1 gene in clinicopathological parameters of Turkish NSCLC patients. Therefore, the study may be improved by increasing the number of patients in the future. Thus, more accurate determinations can be used to define genetic susceptibility.
